# Effects of Extremely Low-Frequency Electromagnetic Field Treatment on ASD Symptoms in Children: A Pilot Study

**DOI:** 10.3390/brainsci14121293

**Published:** 2024-12-22

**Authors:** Kierra Pietramala, Alessandro Greco, Alberto Garoli, Danielle Roblin

**Affiliations:** 1 Leaps and Bounds Exceptional Services ABA (Applied Behaviour Analysis) Program, Leaps and Bounds Clinic, 13045 Jane Street, King City, ON L7B 1A3, Canada; kierra@leapsandboundsservices.com (K.P.);; 2 APSP (Public Agency for Personal Health Services) “Santa Maria”, 38023 Cles, Italy; 3 Departement of Morphology, Surgery and Sperimental Medicine, University of Ferrara, 44121 Ferrara, Italy

**Keywords:** autism, ELF-EMF treatment, applied behavior analysis

## Abstract

Background/Objectives: Autism Spectrum Disorder (ASD) are neurodevelopmental disorders marked by challenges in social interaction, communication, and repetitive behaviors. People with ASD may exhibit repetitive behaviors, unique ways of learning, and different ways of interacting with the world. The term “spectrum” reflects the wide variability in how ASD manifests in individuals, including differences in abilities, symptoms, and support needs, and conditions characterized by difficulties in social interactions, communication, restricted interests, and repetitive behaviors. Inflammation plays a crucial role in the pathophysiology, with increased pro-inflammatory cytokines in cerebrospinal fluid. Previous studies with transcranial magnetic stimulation have shown promising results, suggesting nervous system susceptibility to electromagnetic fields, with evidence indicating that extremely low-frequency electromagnetic field (ELF-EMF) treatment may modulate inflammatory responses through multiple pathways, including the reduction of pro-inflammatory cytokines like IL-6 and TNF-α, and the enhancement of anti-inflammatory mediators. Methods: This pilot study included 20 children (ages 2–13) with a confirmed diagnosis of ASD. A 15-week protocol involved ELF-EMF treatments using the SEQEX device, with specific day and night programs. Assessment was conducted through standardized pre- and post-treatment tests: Achenbach Child Behavior Checklist, Peabody Picture Vocabulary Test-4, Expressive One Word Picture Vocabulary Test-4, and Conner’s 3GI. Results: Statistically significant improvements were observed in receptive language (PPVT-4: from 74.07 to 90.40, *p* = 0.002) and expressive language (EOWPVT-4: from 84.17 to 90.50, *p* = 0.041). Notable reductions, with statistical significance, were found in externalizing problems across both age groups (1.5–5 years: *p* = 0.028; 6–18 years: *p* = 0.027), with particular improvement in attention and behavioral problems. The results were observed over a short period of 15 weeks, therefore excluding the possibility of coincidental age-related gains, that would typically occur during a normal developmental timeframe. Parent evaluations showed significant reduction in ASD symptoms, particularly in the 1.5–5 years group (*p* = 0.046). Conclusions: ELF-EMF treatment demonstrated a high safety profile and efficacy in mitigating ASD-related symptoms. The observed improvements suggest both direct effects on central and autonomic nervous systems and indirect effects through inflammatory response modulation. Further studies are needed to confirm these promising results through broader demographics and randomized control designs.

## 1. Introduction

Autism Spectrum Disorders (ASDs) represent a heterogeneous group of neurobiological conditions characterized by significant difficulties in social interactions and communication, and the presence of restricted interests and repetitive behaviors [1]. Although the prevalence of ASD is steadily increasing [2,3,4,5], the exact causes still remain unclear, and research continues to focus on identifying new biomarkers and optimizing therapeutic interventions. Epidemiological and clinical findings in ASD cannot be explained by the traditional linear genetic model, hence the need to move to a more fluid concept that integrates genetics, environment, and epigenetics as a whole [6]. The fetal period and the first two years of life (the so-called “first 1000 days”) are the crucial time window for neurodevelopment [6]. The interaction between immune activation, gut dysbiosis, and mitochondrial impairment appear to correlate significantly with neurodevelopment [6].

In the field of Integrative Medicine, a recent machine learning study identified how natural compounds like *Hypericum perforatum* can target multiple molecular pathways, particularly through anti-inflammatory mechanisms and immune system modulation, effects that might parallel those of ELF-EMF treatment on the nervous system, as hypothesized in this study [7]. For ASD, while various therapeutic approaches exist, from behavioral interventions to standard pharmacological treatments, there remains a critical need for targeted therapies that can address the complex pathophysiology of the condition, including its inflammatory and immune components.

It is known, however, how inflammation plays a crucial role in the pathophysiology of this disorder [8,9], with increased CSF concentrations of pro-inflammatory cytokines [8] and other dysregulations, such as mitochondrial disfunction [10,11]. Particularly, it is broadly recognized that microglial activation is implicated in the pathophysiology of ASD [8]. Microglia, the resident immune cells of the CNS, are classified within the myeloid lineage, alongside monocytes and macrophages [12]. Unlike peripheral counterparts, microglia originate during early embryogenesis from progenitor cells in the yolk sac, subsequently migrating into the nascent brain [12]. Furthermore, they orchestrate responses to pathogenic insults and injury, engaging in pathogen recognition, antigen presentation, antibody-dependent responses, secretion of reactive oxygen species and cytokines, extracellular matrix remodeling, and regulation of both inflammatory and immune pathways [13]. The concurrent upregulation of iNOS, glutaminase, and inducible cyclooxygenase (COX-2) culminates in increased levels of nitric oxide, glutamate, and prostaglandins, respectively. Collectively, these factors exert deleterious effects on neuronal integrity and viability [12,13]. Following CNS injury, microglial cells undergo an activation state characterized by elevated production and expression of cytokines and chemokines, as well as the induction of nitric oxide (NO) synthesis, by inducible nitric oxide synthase (iNOS) [14,15].

The interaction between immune activation, gut dysbiosis, and mitochondrial impairment appear to correlate significantly with neurodevelopment [11]. Evidence suggests that these factors are interconnected, as mitochondrial dysfunction can lead to increased oxidative stress and inflammation, which in turn may affect brain function and development [10,11]. Studies have shown decreased activity of mitochondrial electron transport chain complexes and reduced gene expression in individuals with ASD, particularly in brain regions associated with cognitive and behavioral features [11]. Additionally, these mitochondrial abnormalities have been linked to immune system dysregulation and gastrointestinal dysfunction, suggesting a complex interplay between these systems in ASD pathophysiology [11].

On the non-pharmacological therapeutic front, there are positive indicators of a response to treatment with transcranial magnetic stimulation [16,17,18]. Over the past decade, non-invasive brain stimulation (NIBS) techniques [19,20,21], including transcranial direct current stimulation (tDCS) [22,23,24] and transcranial magnetic stimulation (TMS) [16,25,26,27,28], have been proposed as potential therapeutic strategies to influence maladaptive neuroplasticity or induce beneficial plastic changes in neuropsychiatric disorders such as ASD [16,17,18,19,20,21,22,23,24,25,26,27,28]. Neuropathological investigations have revealed that patients with ASD exhibit an imbalance between excitatory and inhibitory cortical activity [16,17,18,19,20,21]. The rationale for using noninvasive electromagnetic stimulation in ASD is that facilitating or suppressing specific neuronal populations may restore the cortical excitatory–inhibitory equilibrium, thereby enhancing the associated functional capacities governed by these brain areas [16,17,18,19,20,21,22,23,24,25,26,27,28].

Furthermore, ASD symptoms often co-occur with sleep disturbances and depression, forming a complex interplay that can significantly impact the quality of life for individuals on the spectrum and their families [29]. Disrupted sleep patterns associated with ASD symptoms suggest a neuroinflammatory milieu, with aberrant synaptic regulation by activated microglia, compromising sleep architecture, which might exacerbate depressive symptoms in ASD populations [30]. In a study [30], ASD poor sleepers differed from ASD good sleepers on actigraphic (sleep latency, sleep efficiency, fragmentation) and polysomnographic (sleep latency) measures, and were reported to have more inattention, hyperactivity, and restricted/repetitive behaviors. Fragmentation was correlated with more restricted/repetitive behaviors.

Sleep disturbances in children with autism spectrum disorder (ASD) encompass both macro- and micro-architectural alterations in sleep structure [30,31,32,33,34,35,36]. At the macro level, these disturbances can manifest as prolonged sleep onset latency, increased nighttime awakenings, reduced total sleep time, and alterations in the distribution of sleep stages—particularly reductions in rapid eye movement (REM) sleep and/or atypical slow-wave sleep patterns [31,32]. On a micro-architectural scale, abnormalities may include altered sleep spindle density, irregularities in K-complex formation, and atypical spectral power profiles across sleep stages [33,34]. Collectively, these disturbances suggest a fundamental dysregulation of sleep homeostasis and sleep-dependent processes integral to learning, memory, and emotional regulation [33,34].

Crucially, these altered sleep patterns have implications that extend well beyond nighttime rest, influencing daytime mood, behavior, and overall psychological well-being. Sleep disruption in ASD has been associated with elevated irritability, anxiety, and internalizing symptoms [35,36]. Given that mood regulation is closely intertwined with sleep quality, it follows that persistent macro- and micro-level sleep abnormalities may contribute to, or exacerbate, mood dysregulation and depressive symptoms in this population. Indeed, children with ASD and more pronounced sleep disturbances frequently exhibit greater emotional reactivity, reduced stress tolerance, and an increased prevalence of comorbid mood disorders, including depression.

In ASD, where neuroinflammatory processes, atypical synaptic organization, and sensory sensitivities already pose challenges to emotional regulation, disturbed sleep architecture may further destabilize neural systems responsible for mood homeostasis.

Taking all the aforementioned comorbidities and considering results from a previous experience, carried out using an EMF treatment device developed in Italy (SEQEX), on a small group of children with ASD [37], it was decided to replicate prior findings, based on recent evidence from the application of ELF-EMF therapies. Interventions that improve sleep quality at both the macro and micro levels hold promise not only for enhancing sleep itself but also for mitigating mood disturbances and reducing the risk of depression in children with ASD. This integrative approach underscores the importance of comprehensive management strategies that address the complex interplay between sleep architecture, affective symptoms, and overall quality of life in ASD.

Particularly, the purpose of this pilot study is to investigate the effects of the SEQEX medical device on altered cognition and behavior, and to confirm its impact on decreasing inflammatory markers in the brain and body of individuals affected by ASD, possibly interacting with macro/micro sleep architecture. Given that parents of children with ASD provided substantial experiential evidence of improvements observed, such as better sleep, reduced challenging behaviors, enhanced language skills, and increased cognitive ability, we opted to quantify and corroborate this anecdotal evidence through standardized testing.

## 2. Materials and Methods

This pilot study consisted of 20 participants. It was conducted in the Leaps and Bounds Learning Centre in Ontario, Canada. Meta analysis and formalization of the research protocol and the subsequent data evaluation was carried out by the Italian team, part of the international group that took part in the research (Greco, Garoli).

### 2.1. Study Sample and Recruitment Criteria

This observational study includes only one treatment group, with pre- and post-treatment surveys. It was designed as a 15-week protocol. All children in the pilot study have been diagnosed as affected by ASD. Participants were between 2 and 13 years of age. Individuals without a confirmed ASD diagnosis were obviously excluded; individuals below 2 years of age or older than 13 were excluded.

Sample size was determined based on Crescentini’s pilot study (2007) [37], which first examined effects of ELF-EMF treatment in 8 children with ASD. Given those preliminary positive results, we aimed to expand the sample size while maintaining the feasibility of this pilot investigation. We enrolled 20 participants to account for potential dropouts. After 4 dropouts, the final cohort consisted of 16 participants evenly distributed between age groups (8 children aged 2–5 years and 8 children aged 6–13 years). While this represents a limited sample size, our 15-week protocol with comprehensive assessment battery allowed us to detect meaningful changes in behavioral and physiological measures in this preliminary investigation.

### 2.2. Statistical Evaluation

All children were assessed on pre- (T0), on half- (T1, after 8 weeks) and post- (T2) treatment with SEQEX device, through the following assessments:

1. Achenbach Child Behavior Checklist CBCL [38,39,40] (Forms for 1.5–5 years of age and 6–18 years of age), as rated by both the therapist and the parents. This checklist assesses behavioral and emotional and cognitive concerns as rated by the parents of the subjects.
2. Peabody Picture Vocabulary Test 4th Edition [41,42]. This test measures receptive vocabulary and is considered our measure of “cognitive functioning”.
3. The Expressive One Word Picture Vocabulary Test 4th Edition [43]. This test measures expressive vocabulary and is also considered a measure of “cognitive functioning”.

Children’s scores from the pre-tests to the post-tests from all participants were compared and analyzed with the Achenbach Teacher & Parent Wilcoxon test. In relation to the Peabody Picture Vocabulary Test and the Expressive One Word Picture Vocabulary Test, paired sample tests and calculated effect sizes were completed.

#### Explanation of Metrics Used

**ACHENBACH CBCL/TRF** (Child Behavior Checklist/Teacher Report Form) is broken down into two age groups: 1.5–5 years and 6–18 years.

**CBCL/C-TRF for 1.5–5**-year-old children (Caregiver–Teacher Report Form) [39].

The factor analysis of the scales for the 1.5–5-year-olds combines both boys and girls, as there was no significant difference. The CBCL parent one is based on *N* = 1728 children, and the C-TRF (Teacher) was based on *N* = 1113. They completed test–retest reliability. The correlation (r) was in the 0.80 s and 0.90 s. The mean (m) was 0.85 on the CBCL (Parent) and 0.81 on the C-TRF.

For the internal consistency, Cronbach’s Alpha was used for both referred and non-referred children, and these scores ranged from 0.63 to 0.95. On the CBCL (Parent), the Internalizing scale Cronbach’s Alpha is 0.89, the Externalizing scale Cronbach’s Alpha is 0.92, and the Total Problems scale is 0.95.

On the C-TRF, the Cronbach Alpha ranges from 0.52 to 0.97, the Internalizing scale is 0.89, the Externalizing scale is 0.96, and the Total Problems scale is 0.97.

From the manual for the ASEBA preschool forms and profiles: “*The criterion-related validity of the problems scales was supported by concurrent and predictive associations with a variety of other measures…*” (p. 100)

**CBCL/TRF 6–18-year**-old individuals [40].

The Internal Consistency Cronbach Alpha for the CBCL ranges from 0.72 to 0.97. The Internalizing scale is 0.90, the Externalizing scale is 0.94, and Total Problems scale is 0.97.

On the TRF, the Cronbach Alpha ranges from 0.73 to 0.97. The Internalizing scale is 0.90, the Externalizing scale is 0.95, and the Total Problems scale is 0.97.

The ASEBA school-aged forms and profiles manual indicates “the criterion-related validity of the CBCL, YSR and TRF scales was supported by multiple regressions, odds ratios, and discriminant analyses all of which showed significant (*p* < 0.01) discrimination between referred and nonpreferred children” (p. 135). “*The construct validity of the scales has been supported in many ways, such as evidence for significant associations with analogous scales of other instruments and with DSM criteria*.” (p. 135)

**PPVT-4** (Peabody Picture Vocabulary Test 4th Edition) [42].

Pearson Assessments.

Reliability: *N* = 3540

Split half & Coefficient Alpha: “*Split-half reliability and coefficient alpha of each form were calculated for each of the 28 age groups in the age norm sample and for each of the 13 groups in the grade norm sample*” (p. 53). We used the age norms. The Split-Half reliability for the age norms is 0.94. The Alpha for form A is 0.97 and for form B is 0.96.

Alternate-Form Reliability: *N* = 508. Ranges from 0.87 to 0.93 with a mean of 0.89.

Test-Retest Reliability: *N* = 340. Test-retest correlation of 0.93 (Range 0.92 to 0.96).

“*The various types of reliability evidence presented in this chapter indicate that PPVT-4 scores are highly precise; that is they are affected only minimally by sources of measurement error such as content sampling, intervening learning, and difficulties in the examinee’s physical or emotional state*” (p. 56).

Content Validity: “*Stimulus words were selected from a pool of words that could be illustrated by color drawings that represented 20 content areas. The pool consisted primarily of entries in Marriam-Webster’s Collegiate Dictionary (2003) and various editions of Webster’s New Collegiate Dictionary (1953, 1967, 1981*)” (p. 58).

Standardized from ages 2 years 6 months to 81 years of age. *N* = 3540 for Age norms.

“*PPVT-4 norm sample consists of U.S. residents aged 2 years 6 months and older who were proficient in English and did not meet any of the preestablished exclusionary criteria… at each age and grade, the sample was intended to match the U.S. population with respect to sex, race/ethnicity, SES, geographic region and special-education status.*” (p. 33)

**EOWPVT-4** (Expressive One-Word Picture Vocabulary Test, Fourth Edition) [43].

Academic Therapy Publications.

Reliability:

Ranging from 0.93 to 0.97, with a median of 0.95.

Cronbach’s Alpha *N* = 2394: “*obtained at each age level, as well as overall ages”. “Computed by age group for all individuals participating in the standardization study”* (p. 53).

Validity:

Content Validity: “*the EOWPVT-4 format allows an examinee to demonstrate his or her vocabulary ability in a way consistent with academic and everyday tasks.”* (p. 57)

Construct Validity: Correlation between the WISC-4 Verbal Comprehension Index (VCI) & EOWPVT-4 is 0.43 (*N* = 23)

Criterion Related Validity:

Assumptions that underlie the EOWPVT-4 (quoted from p. 59 of the manual):

(1) *Vocabulary is an ability that is developed over time, with exposure to a variety of sources (home, academic, occupational)*
(2) *Vocabulary is related to one’s reading ability*
(3) *Vocabulary can be related to one’s cognitive ability*
(4) *Vocabulary scores could be expected to be lower in persons with known disabilities*

Reading: *N* = 33, correlation of 0.69 between EOWPVT-4 and STAR Reading standard scores.

Cognition: *N* = 24, correlation of 0.35 between EOWPVT-4 and WISC-4 FSIQ.

Exceptional Groups: “*EOWPVT-4 scores were significant lower in those who had diagnoses of ADD (*N* = 39), LD (*N* = 74), Reading Disability (*N* = 53), Autism (*N* = 28), and Specific Language Impairment (*N* = 14) than those without diagnoses. All differences were significant at the 0.001 level.* (p. 61).”

### 2.3. ELF-EMF SEQEX Treatment

A SEQEX^®^ device was used for the study. This device is produced and distributed by the Italian company S.I.S.T.E.M.I. Srl (Trento, Italy), which is certified CSQ ISO-13485. These devices produce complex electromagnetic fields using an analog mechanism, operating in a frequency range from 1 to 80 Hz and at variable intensities from 1 to 20 µT. The field parameters were tested by the manufacturing company using specialized equipment: a GM08 Gaussmeter produced by the Hirst company. The electromagnetic field produced by the device’s control unit—on which the parameters of the electromagnetic field are set—is emitted from a mat containing a Helmholtz coil that generates the ELF-EMF. Individual patients were asked to lie on the mat to receive non-focused, total-body treatment with weak electromagnetic fields.

Studies found that macro/micro sleep architecture seems to be extremely significant in the exacerbation of ASD symptomatology [44]. Insomnia and daytime behavioral problems are common issues in pediatric ASD, yet specific underlying relationships with Non-Rapid Eye Movement sleep (NREM) and Rapid Eye Movement (REM) sleep architecture are understudied [44]. REM sleep alterations (REM%, REM EEG power) could be associated with more internalizing behaviors and NREM sleep deficits (N3%; slow wave activity (SWA) 1–3 Hz EEG power) could be linked to increased externalizing behaviors in children with ASD [44].

Since the lower frequency range (1–20 Hz) overlaps with the frequency range of the most powerful spectral components of the EEG signal emitted by the brain during NREM sleep, it seems to be probable that a 1 Hz EMF, close to natural intensity, might interfere with human sleep. Particularly, an increased amount of the deepest sleep stage (N3), distinguished from other stages by high amplitude, would be an indicator of neuro-restorative functions. In a study [45], an increase in N3 and other NREM sleep stages was obtained by exposing 23 healthy volunteers during the 10–50th minutes of an afternoon napping attempt to a low-level (0.004 μT) 1-Hz EMF. Although the amount of N3 remained unchanged in this exposure condition, the total duration of sleep became longer, due to an increase in the N2 amount.

The effect of the 1 Hz/0.004 μT electromagnetic field exposure on stage N3 was not significant despite the overlap of this intervention frequency with that of slow waves. However, the total duration of sleep was significantly increased due to a noteworthy increase in the stage N2 amount. The exposure to the 1 Hz electromagnetic field did not reveal any sleep-disturbing effects; instead, it increased the total duration of sleep, due to the increase in the N2 amount. This result suggests a possibility of sleep-promoting action of 1 Hz EMF exposure.

As sleep measures reflect concerning externalizing behaviors in ASD and could serve as a biomarker for mood disorders in the ASD population, the authors devised a protocol architecture that could ensure both nocturnal and diurnal EMF exposure. In the previous Italian study [37], it was found that fixed-time exposures potentially caused some unexpected side effects (increased aggressivity possibly due to stress). Henceforth, in this current study, incremental time-use was designed to facilitate children’s collaboration and to allow familiarization with the procedure. We devised a weekly pattern of both nighttime (program D) and daytime (program AUT) use:

1. Week 1: D Monday, Friday; AUT Wednesday, Sunday
2. Week 2: D Monday, Wednesday, Friday; AUT Tuesday, Thursday, Saturday
3. Week 3: D Tuesday, Thursday, Saturday; AUT Monday, Wednesday, Friday, Sunday
4. Week 4: D Tuesday, Thursday, Saturday; AUT every day except Thursday
5. Week 5: D Monday, Tuesday, Thursday, Saturday, Sunday; AUT every day except Thursday
6. Week 6: D every day except Wednesday; AUT every day
7. Week 7–15: D and AUT every day.

Environmental factors were standardized across participants. In order to reduce additional stress and to minimize confounding variables caused by travel, car use, and changes in environment, we concluded that treatments could be administered by the participants’ parents in the comfort and familiar space of their homes, albeit under a strict protocol structure. Time-use (180 min for program D and 27 min for program AUT), daily time of administration (2 p.m. for AUT and around 9 p.m. after falling asleep), and quality of the environment (reduced noises and stable home temperatures) were fixed for all participants.

The **D** night program is defined as in the Table 1. This simulates Delta brain rhythm frequencies, characteristic in the NREM sleep phase, in order to intervene on sleep disturbances, typical of ASD [44,45,46,47].

The **AUT** daytime program is defined as in Table 2. The sequence of frequencies in this program includes a set studied to produce several effects. A stimulating effect on the central nervous system (frequency: 1 Hz [48,49], 10 Hz [50,51], 12 Hz [52], 20 Hz [49], 50 Hz [53,54,55]); an overall antioxidant stimulus (freq: 4 Hz [56], 50 Hz [57]); a mediatory function on the enteric system (frequency: 8 Hz [58]); a general anti-inflammatory effect (frequency: 2 Hz [59], 50 Hz [60,61,62,63], 75 Hz [64]). All these sets have been validated by research as per specific effect.

## 3. Results

Twenty participants were enrolled, with four dropouts. Eight participants were in the 2–5 age group and eight participants in the 6–13 age group. The average/mean age of our participants in our study was 5.88 years old.

### 3.1. Peabody Picture Vocabulary Test-4 (PPVT-4) (15 Participants Tested)

**Figure 1:**
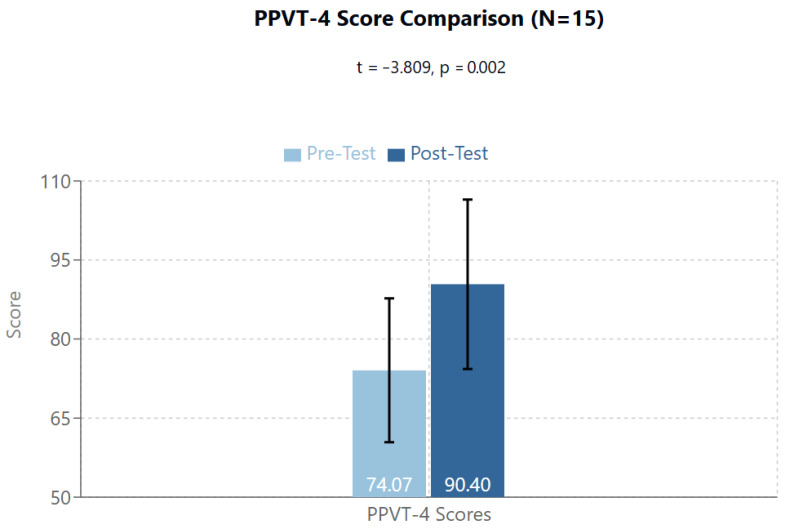
The Peabody Picture Vocabulary Test-4 shows significant difference between Pre- and Post-test t = −3.809, *p* = 0.002.

The T1/Pre-Test group mean was 74.07 (95%CI: 60.43–87.70; SD: 24.62); the T2/Post-Test group mean was 90.40 (95%CI: 74.33–106.47; SD: 29.01). The T1/Pre-Test to T2/Post-Test scores emerged as highly statistically significant (t = −3.809, *p* = 0.002) (Figure 1).

### 3.2. Expressive One Word Picture Vocabulary Test-4th Edition (EOWPVT-4) (15 Participants Tested)

**Figure 2:**
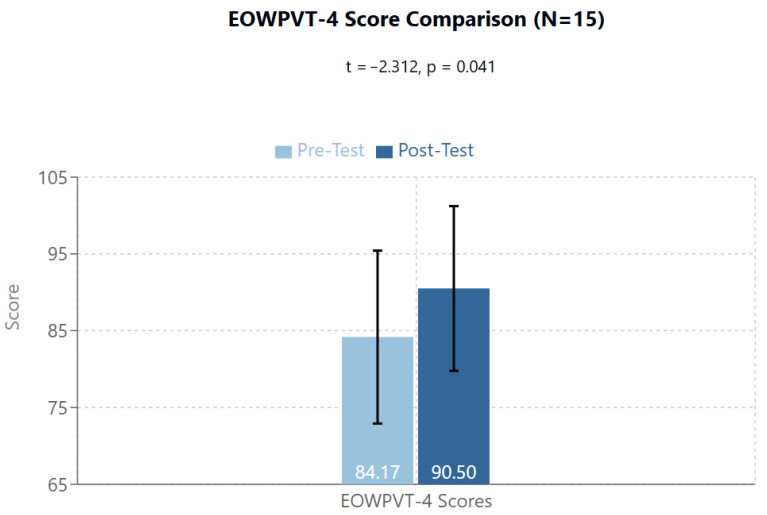
The Expressive One Word Picture Vocabulary Test-4th Edition shows significant difference between Pre- and Post-Test t = −2.312, *p* = 0.041.

The T1/Pre-Test group mean was 84.17 (95%CI: 72.92–95.41; SD: 17.69); T2/Post-Test group mean was 90.50 (95%CI: 79.78–101.22; SD: 16.86). The T1/Pre-Test to T2/Post-Test scores emerged as statistically significant (t = −2.312, *p* = 0.041) (Figure 2).

### 3.3. Achenbach Teacher Data Ages 1.5–5 Years (8 Participants Tested)

The Internalizing Area did not show statistical significance.

**Figure 3:**
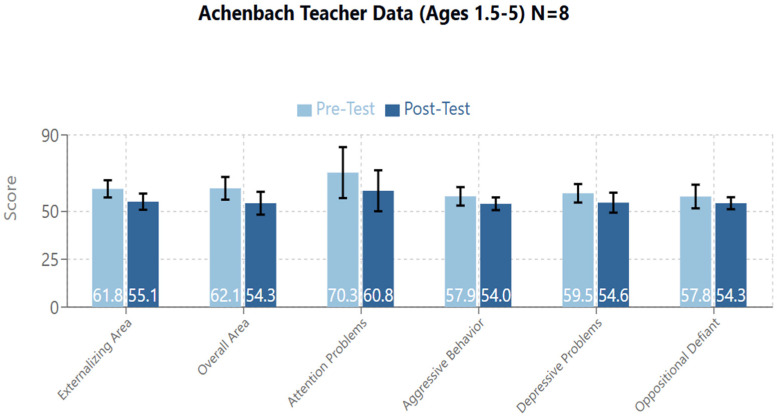
Cumulative graph for areas with significant results in the Achenbach Teacher Data for participants from 1.5 to 5 years.

The Externalizing Area and Overall (Total Problems) score emerged as statistically significant (Figure 3, Table 3).

- Externalizing Area: T1/Pre-Test mean = 61.8 (95%CI: 57.31–66.2; SD: 5.31), T2/Post-Test mean = 55.1 (95%CI: 50.87–59.37; SD: 5.08), Standardized Test Statistic: −2.201, *p* = 0.028. There were 6 Negative Differences, 1 Positive Difference, and 1 Tie. Therefore, 6 of 8 participants showed a decrease on their score in this area.
- Overall (Total Problems) Area: T1/Pre-Test mean = 62.1 (95%CI: 56.17–68.08; SD: 7.12), T2/Post-Test mean = 54.3 (95%CI: 48.32–60.17; SD: 7.08), Standardized Test Statistic: −2.383, *p* = 0.017. There were 7 Negative Differences and 1 Positive Difference. Therefore, 7 of 8 participants saw a decrease on their score in this area.

The following scales on the Internalizing area did not show statistical significance: Emotionally Reactive, Anxious/Depressed, and Somatic Complaints.

Within the Internalizing Area, the Withdrawn scale emerged as not significant. T1/Pre-test mean = 59.3 (95%CI: 52.9–65.6; SD: 7.55), T2/Post-Test mean = 57.3 (95%CI: 50.99–63.5; SD: 7.48), Standardized Test Statistic: −1.784, *p* = 0.074. There were 5 Negative Differences, 2 Positive Differences and 1 Tie. Therefore, 5 of 8 participants saw a decrease on their score in this area.

In the Externalizing Area, both Attention Problems & Aggressive Behavior scales emerged as statistically significant.

- Attention Problems: T1/Pre-Test mean = 70.3 (95%CI: 56.99–83.51; SD: 15.86), T2/Post-Test mean = 60.8 (95%CI: 50.11–71.4; SD: 12.73), Standardized Test Statistic: −2.366, *p* = 0.018. There were 7 Negative Differences and 1 Tie. Therefore, 7 of 8 participants saw a decrease in their score on this area.
- Aggressive Behavior: T1/Pre-Test mean = 57.9 (95%CI: 53.07–62.68; SD: 5.74), T2/Post-Test mean = 54.0 (95%CI: 50.68–57.31; SD: 3.96), Standardized Test Statistic: −2.205, *p* = 0.027. There were 6 Negative Differences, 1 Positive Difference, and 1 Tie. Therefore, 6 of 8 participants saw a decrease in their score in this area.

The following scales on the DSM-5 Oriented scales the following scales emerged as statistically significant: Depressive Problems and Oppositional Defiant Problems.

- Depressive Problems: T1/Pre-Test mean = 59.5 (95%CI: 54.67–64.33; SD: 5.78), T2/Post-Test mean = 54.6 (95%CI: 49.38–59.87; SD: 6.28), Standardized Test Statistic: −1.983, *p* = 0.047. There were 6 Negative Differences, 1 Positive Difference, and 1 Tie. Therefore, 6 of 8 participants saw a decrease in their scores on this area.
- Oppositional Defiant Problems: T1/Pre-Test mean = 57.8 (95%CI: 51.61–63.89; SD: 7.34), T2/Post-Test mean = 54.3 (95%CI: 51.16–57.34; SD: 3.69), Standardized Test Statistic: −1.992, *p* = 0.046. There were 5 negative differences, 1 positive difference, and 2 ties. Therefore, 5 of 8 participants saw a decrease in their score on this area.

The ASD symptom scale emerged as marginally significant. T1/Pre-Test mean = 65.5 (95%CI: 56.03–74.97; SD: 11.33), T2/Post-Test mean = 58.1 (95%CI: 51.58–64.67; SD: 7.83), Standardized Test Statistic: −1.897, *p* = 0.058. There were 5 Negative Differences, 1 Positive Difference, and 2 Ties. Therefore, 5 of 8 participants saw a decrease on their scores in this area.

On the DSM-5 Oriented Scales, the following scales did not show statistical significance: Anxiety Problems, and Attention Deficit/Hyperactivity Problems.

### 3.4. Achenbach Teacher Data Ages 6–18 Years (7 Participants Tested)

**Figure 4:**
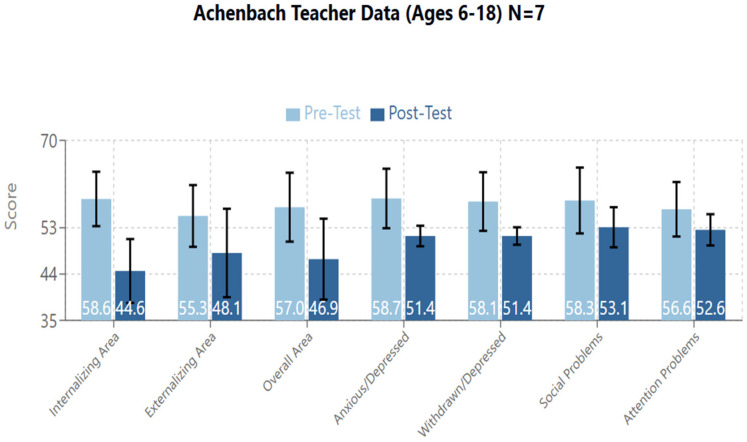
Cumulative graph for areas with significant results in the Achenbach Teacher Data for participants aged 6–18.

The following areas all showed statistical significance: The Internalizing Area, Externalizing Area, and Overall (Total Problems) score (Figure 4, Table 4).

- Internalizing Area: T1/Pre-Test mean = 58.6 (95%CI: 53.31–63.83; SD: 5.68), T2/Post-Test mean = 44.6 (95%CI: 38.4–50.75; SD: 6.68), Standardized Test Statistic: −2.366, *p* = 0.018. There were 7 Negative Differences. Therefore, all 7 participants saw a decrease in their score on this area.
- Externalizing Area: T1/Pre-Test mean = 55.3 (95%CI: 49.3–61.3; 6.49), T2/Post-Test mean = 48.1 (95%CI: 39.5–56.8; SD: 9.35), Standardized Test Statistic: −2.214, *p* = 0.027. There were 6 Negative Differences and 1 Tie. Therefore, 6 of 7 participants saw a decrease in their score on this area.
- Overall (Total Problems) Area: T1/Pre-Test mean = 57.0 (95%CI: 50.3–63.71; SD: 7.26), T2/Post-Test mean = 46.9 (95%CI: 39.04–54.68; 8.45), Standardized Test Statistic: −2.375, *p* = 0.018. There were 7 negative differences. Therefore, all 7 participants saw a decrease in their score on this area.

Within the Internalizing Area, the scores on the following scales emerged as statistically significant: Anxious/Depressed and Withdrawn/Depressed.

- Anxious/Depressed: T1/Pre-Test mean = 58.7 (95%CI: 52.92–64.51; SD: 6.26), T2/Post-Test mean = 51.4 (95%CI: 49.4–53.5; SD: 2.23), Standardized Test Statistic: −2.201, *p* = 0.028. There were 6 Negative Differences and 1 Tie. Therefore, 6 of 7 participants saw a decrease on their scores in this area.
- Withdrawn/Depressed: T1/Pre-Test mean = 58.1 (95%CI: 52.4–63.9; SD: 6.23), T2/Post-Test mean = 51.4 (95%CI: 49.7–53.1: 1.81), Standardized Test Statistic: −2.214, *p* = 0.027. There were 6 Negative Differences and 1 Tie. Therefore, 6 of 7 participants saw a decrease on their score in this area.

Within the Internalizing Area, the following score did not show statistical significance: Somatic Complaints.

Within the Externalizing Area the following scores showed statistical significance: Social Problems and Attention Problems.

- Social Problems: T1/Pre-Test mean = 58.3 (95%CI: 51.9–64.65; SD: 6.87), T2/Post-Test mean = 53.1 (95%CI: 49.2–57.1; 4.26), Standardized Test Statistic: −2.023, *p* = 0.043. There were 5 Negative Differences and 2 Ties. Therefore, 5 of 7 participants saw a decrease on their scores in this area.
- Attention Problems: T1/Pre-Test mean = 56.6 (95%CI: 51.3–61.9; SD: 5.74), T2/Post-Test mean = 52.6 (95%CI: 49.56–55.6; SD: 3.26), Standardized Test Statistic: −2.201, *p* = 0.028. There were 6 Negative Differences and 1 Tie. Therefore, 6 of 7 participants saw a decrease on their scores in this area.

Within the Externalizing Area, the Thought Problems scale score emerged as marginally significant. T1/Pre-Test mean = 58.1 (95%CI: 50.8–65.5; SD: 7.97), T2/Post-Test mean = 55.3 (95%CI: 48.69–61.88; SD: 7.13), Standardized Test Statistic: −1.890, *p* = 0.059. There were 4 Negative Differences and 3 Ties. Therefore, 4 of 7 participants saw a decrease in their scores on this area.

Within the Externalizing Area, the following scores did not show statistical significance: Rule Breaking Behavior and Aggressive Behavior.

**Figure 5:**
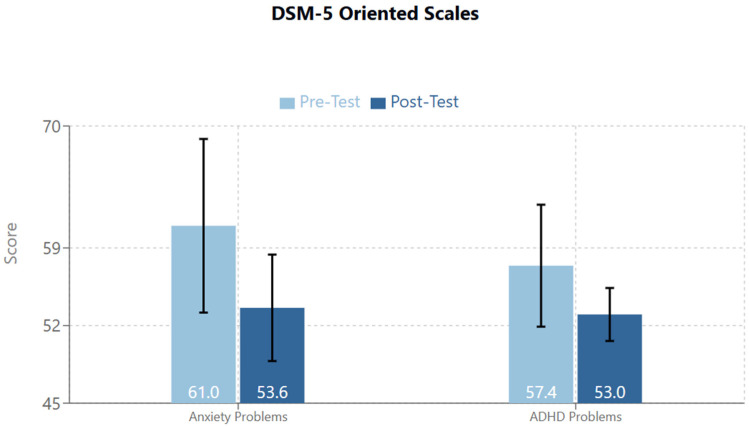
The DSM−5 Oriented Scales showed significant changes in the areas of anxiety disorders (*p* = 0.027) and attention disorders (*p* = 0.042).

On the DSM-5 Oriented Scales, the following scales demonstrated statistical significance: Anxiety Problems and Attention Deficit/Hyperactivity Problems (Figure 5).

- Anxiety Problems: T1/Pre-Test mean = 61.0 (95%CI: 53.17–68.83; SD: 8.47), T2/Post-Test mean = 53.6 (95%CI: 48.8–58.34; SD: 5.16), Standardized Test Statistic: −2.207, *p* = 0.027. There were 6 Negative Differences and 1 Tie. Therefore, 6 of 7 participants saw a decrease in their score in this area.
- Attention Deficit/Hyperactivity Problems: T1/Pre-Test Mean = 57.4 (95%CI: 51.9–62.9; SD: 5.88), T2/Post-Test Mean = 53.0 (95%CI: 50.61–55.4; SD: 2.58), Standardized Test Statistic: −2.032, *p* = 0.042. There were 5 Negative Differences and 2 Ties. Therefore, 5 of 7 participants saw a decrease on their scores in this area.

On the DSM-5 Oriented Scales, the following scales did not show statistical significance: Depressive Problems, Somatic Problems, and Conduct Problems.

Oppositional Defiant Problems emerged as marginally significant. T1/Pre-Test mean = 57.9 (95%CI: 49.5–66.2; SD: 9.1), T2/Post-Test mean = 53.7 (95%CI: 47.6–59.8; SD: 6.58), Standardized Test Statistic: −1.841, *p* = 0.066. There were 4 Negative Differences and 3 Ties. Therefore, 4 of 7 participants saw decreases on their scores in this area.

### 3.5. Achenbach Parent Data Ages 1.5–5 Years (6 Participants Tested)

The following areas did not show statistical significance: The Internalizing Area, Externalizing Area, and Overall (Total Problems) score.

The following scales within the Internalizing Area did not show statistical significance: Emotionally Reactive, Anxious/Depressed, Somatic Complaints, and Withdrawn.

The following scales within the Externalizing Area did not show statistical significance: Attention Problems and Aggressive Behavior.

Within the Externalizing area, the Aggressive Behavior scale emerged as marginally significant. (T1/Pre-Test mean = 57.0 (95%CI: 57–52.2; SD: 6), T2/Post-Test mean = 52.2 (95%CI: 49.6–54.8; SD: 2.5), Standardized Test Statistic: −1.826, *p* = 0.068). There were 4 Negative Differences, 0 Positive Differences and 2 Ties. Therefore, 4 of 6 participants saw a decrease in their overall score on this scale.

Not loading on either the Internalizing or Externalizing areas, the “Sleep Problems” scale did not emerge as statistically significant.

**Figure 6:**
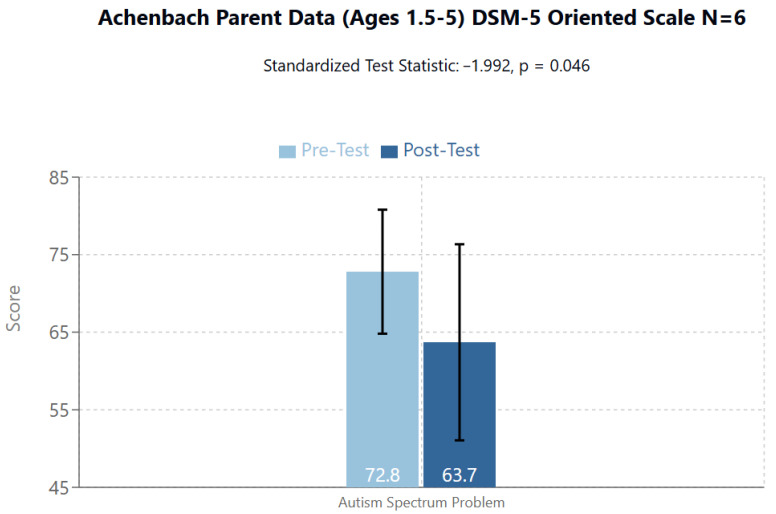
In the 1.5–5 age group, the Achenbach Parent Data DMS-5 Oriented Scale showed significant improvement in the ASD item.

Significant Score on the DSM-5 Oriented Scales (Figure 6):

- The ASD scale from T1/Pre-Test to T2 Post-Test emerged statistically significant (T1/Pre-Test mean = 72.8 (95%CI: 64.8–80.9; SD: 7.68), T2/Post-Test mean = 63.7 (95%CI: 51.06–76.3; SD: 12.0), Standardized Test Statistic: −1.992, *p* = 0.046). There were 5 Negative Differences and 1 Positive Difference. There for 5 of 6 participants saw a decrease in their overall score on this scale.

On the DSM-5 Oriented Scales, the following scales did not show statistical significance: Depressive Problems, Anxiety Problems, and Oppositional Defiant Problems.

On the DSM-5 Oriented Scales, the Attention-Deficit/Hyperactivity Problems scale emerged as marginally significant. (T1/Pre-Test mean = 56.8 (95%CI: 49.9–63.8; SD: 6.6), T2/Post-Test mean = 53.0 (95%CI: 48.4–57.6; SD: 4.4), Standardized Test Statistic: −1.826, *p* = 0.068). There were 4 Negative Differences and 2 Ties. Therefore, 4 of 6 participants saw a decrease in their overall score on this scale.

### 3.6. Achenbach Parent Data Ages 6–18 Years (7 Participants Tested)

The following areas did not show statistical significance: The Internalizing Area, Externalizing Area, and Overall score.

**Figure 7:**
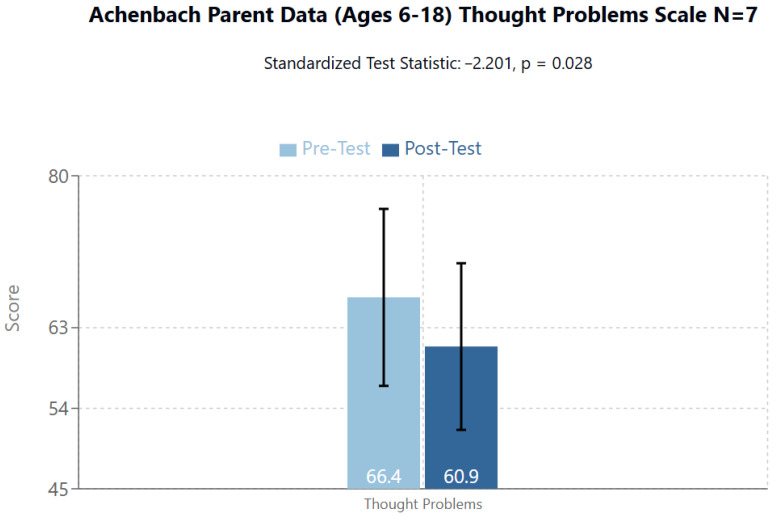
Achenbach Parent Data shows significant difference between Pre- and Post-Test in Thought Problems (t = −2.201, *p* = 0.028).

Significant Score Within the Internalizing area (Figure 7):

Within the Internalizing area, the Thought Problems scale from T1/Pre-Test to T2/Post-Test emerged statistically significant (T1/Pre-Test mean = 66.4 (95%CI: 56.51–76.34; SD:10.7), T2/Post-Test mean = 60.9 (95%CI: 51.59–70.13; 10.0), Standardized Test Statistic: −2.201, *p* = 0.028). There were 6 Negative Differences and 1 Positive Difference. Therefore; 6 of 7 participants saw a decrease in their overall score on this scale.

The following scales within the Internalizing Area did not show statistical significance: Anxious/Depressed, Withdrawn/Depressed, and Somatic Complaints

The following scales within the Externalizing Area did not show statistical significance: Social Problems, Attention Problems, Rule Breaking Behavior, and Aggressive Behavior.

On the DSM-5 Oriented Scales, no scores on any of the clinical scales emerged as statistically significant. These scales are: Depressive Problems, Anxiety Problems, Somatic Problems, Attention-Deficit/Hyperactivity Problems, Oppositional Defiant Problems, and Conduct Problems.

## 4. Discussion

The results obtained demonstrate a significant and encouraging impact of the treatment on various areas of functioning, suggesting a multidimensional therapeutic effect. The first key point to highlight is that none of the treated subjects experienced any side effects from the ELF-EMF treatment. However, it is important to acknowledge that this preliminary study was conducted with a small sample size, which may limit the generalizability of the findings. Despite this limitation, the absence of adverse effects in the treated group is a promising indicator of the treatment’s safety profile. This initial finding is crucial for determining the feasibility of further research and provides reassurance for families who have already begun or plan to initiate EMF treatment integration for their children.

Given the small sample size, future studies should aim to include a larger and more diverse population to validate these preliminary results and enhance their generalizability. Additionally, expanding the sample size would allow for more robust statistical analyses, enabling researchers to identify potential moderators and mediators of treatment efficacy. Further investigation is also warranted to explore the long-term effects of ELF-EMF treatment, assess its impact across different subgroups within the ASD population, and compare its efficacy with other established interventions. By addressing these areas, subsequent research can build upon the foundational insights provided by this study, ultimately contributing to more comprehensive and evidence-based therapeutic strategies for children with ASD.

An important initial effect observed was relative to language skills. Specifically, significant improvements were noted in both receptive (PPVT-4: from 74.07 to 90.40, *p* = 0.002) and expressive (EOWPVT-4: from 84.17 to 90.50, *p* = 0.041) language abilities. The improvement in PPVT-4 scores, with a mean increase of 16.33 points (from 74.07 to 90.40), represents a substantial change in receptive vocabulary skills. This increase was statistically significant (t = −3.809, *p* = 0.002) and, occurring over a relatively short 15-week period, suggests a meaningful enhancement in language comprehension abilities that could impact daily communication and learning capabilities. The robust validation of the PPVT-4 test supports the reliability of these observed changes in receptive language function. The magnitude of change was particularly pronounced in receptive vocabulary, suggesting that the treatment might primarily influence the mechanisms of language input processing. In light of these results and the effects previously demonstrated in a precedent study conducted in Italy on healthy subjects measuring EEG effect of non-focused ELF-EMF exposure [65], we hypothesize a direct effect on the primary auditory areas (temporal lobes), with possible improved functioning of the connectivity between the primary auditory area and language regions—Wernicke’s area for receptive language and Broca’s area for productive language.

This hypothesis is supported by several lines of evidence from electromagnetic field research. Studies have shown that low-frequency magnetic fields can significantly modulate neurotransmitter systems, particularly increasing turnover of dopamine and serotonin in the frontal cortex [51,66]. Additionally, ELF-EMF exposure has been shown to alter cortical excitability through effects on glutamatergic neurotransmission [67], which plays a key role in synaptic plasticity and neural circuit function. The enhanced connectivity between auditory and language regions may be mediated through these neuromodulatory effects, as well as through the demonstrated ability of pulsed electromagnetic fields to influence functional brain networks [65]. Particularly relevant is the finding that ELF-EMF stimulation in the delta range (1–3 Hz) can produce widespread increases in beta band activity across multiple cortical regions, suggesting enhanced neural synchronization and information transfer between brain areas [65]. This increased neural synchrony between temporal and frontal regions could facilitate more efficient processing and integration of auditory and linguistic information. Moreover, the observed improvements may also relate to ELF-EMF’s effects on neuroinflammation and oxidative stress [66], as reduction of inflammatory mediators could optimize synaptic function in language-relevant neural circuits.

The effects obtained on treated subjects were found to be most manifest on so-called externalizing problems in both groups of children divided by age (1.5–5 years: from 61.8 to 55.1, *p* = 0.028; and 6–18 years: from 55.3 to 48.1, *p* = 0.027). In the younger children’s group in particular, there was evidence of improvement in attentional problems (from 70.3 to 60.8, *p* = 0.018) with reduction of aggressive behavior (from 57.9 to 54.0, *p* = 0.027).

In contrast, in the group of older children, a reduction in social (from 58.3 to 53.1, *p* = 0.043) and attentional problems (from 56.6 to 52.6, *p* = 0.028) was observed.

Understanding the differences in externalizing problem scores between younger (1.5–5 years) and older (6–18 years) children with ASD is crucial for tailoring future effective interventions. Externalizing problems typically include behaviors such as aggression, hyperactivity, and oppositional actions. The observed variability in these scores between age groups can be attributed to a combination of developmental, environmental, and intervention-related factors. In younger children, limited cognitive and emotional regulation development can lead to more overt externalizing behaviors. Early intervention can capitalize on neuroplasticity, leading to more significant behavioral improvements; particularly, the D night program simulates and probably reinforces Delta brain rhythm frequencies, characteristic in the NREM sleep phase, naturally more prominent in younger healthy children, especially during deep sleep stages. Older children (6–18) may develop entrenched neural pathways that make behavioral change more challenging, although neuroplasticity remains still active.

In view of the results obtained in different studies conducted in Italy [65,68,69,70], it is possible to hypothesize a direct effect on cortical areas, and especially a modulatory effect on brain electrical activity. In fact, a key hypothesis of the neurobiology of autism spectrum disorder (ASD) is that cortical excitatory function is not sufficiently balanced by inhibitory forces [71,72]. As a pathophysiological alteration of cortical excitatory–inhibitory balance, a direct impact on the generation of EEG oscillations is obvious. To corroborate this point, we analyzed a study [73] that investigated premotor–parietal cortical physiology associated with ASD symptomatology. Behavioral and neurophysiological changes have been reported after exposure to ELF-EMF. The study [73] tested cortical excitability in healthy volunteers. Intracortical facilitation produced by ELF-EMF exposure was significantly enhanced, with an increase of about 20%, while other parameters of cortical excitability remained unchanged. Sham field exposure produced no effects. Effects could be related to cortical glutamatergic activity, generating an enhancement in cortical excitatory neurotransmission. These studies suggests that PEMFs may produce functional changes in the human brain.

In parallel, improved modulation on the limbic system could be the cause of a reduction in aggressive behavior. The effect may have been induced by the modulatory activity of ELF with a general improved control of impulses and reactivity to external arousals [74,75,76,77,78,79,80].

In general, a beneficial effect produced by the multi-level activity can be hypothesized, which could be summarized as:

1. Neurological level:
  a. Improved integration between cortical and subcortical systems.
  b. Enhancement of inhibitory control circuits.
  c. Optimization of sensory processing.
2. Behavioral Level:
  a. Reduction of reactivity to disturbing stimuli.
  b. Improved ability to maintain attention (mediating Beta waves).
  c. Improved control through cortical mediation.
3. Functional Level:
  a. Stress reduction.
  b. Improved social cues recognition and relative interactions.
  c. Increased environmental adaptability.

Regarding ASD symptomatology assessed using the DSM-5 criteria in the 1.5–5-year age group, the most significant finding emerges from the parents’ assessments, which report a significant reduction in ASD problems (from 72.8 to 63.7, *p* = 0.046). This finding is supported by a trend toward significance observed in the teachers’ evaluations (from 65.5 to 58.1, *p* = 0.058). The consistency of improvements across different evaluators (teachers and parents) and across different age groups suggests a robust treatment effect, with the response appearing more pronounced in behavioral and attentional areas than in emotional and somatic areas. The responder rate is generally high, with 5–7 out of 7–8 participants showing improvements on most scales. The generally better results from the parent assessments could be explained by several factors:

- home environment, generally more familiar for children with ASD.
- interaction with parents, usually more natural and instinctive.

Familiarity and comfort in the home environment is an important candidate to explain different findings. Children are typically more relaxed and exhibit their true behaviors at home. The safekeeping aspect of a familiar environment can facilitate more genuine interactions and behaviors, making improvements more noticeable to parents. Schools and other educational settings appear more structured and may present challenges that do not exist at home, potentially masking improvements. These differences could also include temperature shifts, travel time, and stressors such as needing do be on time for appointments, unknown smells, and unknown people’s pheromones or allomones. With parents at home, children with ASD may be exposed to fewer external stressors such as unfamiliar environment, disciplinary measures, and the structured nature of institutes. At home, parents are an integral part of the treatment process and express dynamics of affections (physical contact, touching, expressions, voices) that might not be possible for the teachers. Moreover, parents typically interact with their children multiple times a day, providing numerous opportunities to observe and reinforce positive behaviors and track improvements, while teachers interact with each child for very limited periods, which may not capture the full extent of daily developments. At home, parents might notice gradual progress that might not appear clearly in shorter observation windows.

These latter results can also be considered in light of the work previously produced by the Italian group [65,68,69,70].

As already hypothesized in regard to attentional problems, the effect on the central nervous system (CNS) could be ascribed to a direct effect on frontal lobes and fronto-temporal areas, in addition to an effect on mirror neuron circuits. ELF-EMF treatment may indeed act through neurobiological mechanisms that simultaneously mediate several areas of functioning, with particular impact on attentional, language, and behavioral processes. In a dated study [81], authors specifically showed that low-frequency magnetic field exposure increased both DA and 5-HT turnover in rat frontal cortex. In summary, low-frequency magnetic field exposure has been found to alter both turnover and receptor reactivity of monoaminergic systems, and some behaviors induced by these systems or their agonists and antagonists. We propose that ELF-EMF may also influence the activity of neurotransmitter systems and cholinergic neurons. The overall altered firing patterns in these associated neurotransmitter neural circuits can account for a variety of behavioral alterations produced by ELF-EMF. Lai et al. (1993) [81] already indicated that low-frequency magnetic fields increased the activity of the parasympathetic nervous system, as indicated by increased choline uptake in the frontal cortex and hippocampus, along with enhanced vagal nerve activity. Others [82], using magnetic fields with different parameters (50 Hz, 46 mT), reported decreased irritability.

Moreover, studies have reported hypoactivation in Mirror Neuron System (MNS) regions during social tasks in individuals with ASD. Altered connectivity between MNS regions and other brain areas may disrupt the integration of social information [83,84,85,86,87,88,89,90,91]. Dapretto et al. (2006) [83] utilized functional MRI to demonstrate reduced activation in the Inferior Frontal Gyrus (IFG), a key Mirror Neuron region during imitation tasks, in children with ASD compared to typically developing controls. Recognized areas involved in Mirror Neuron activities are (a) the Inferior Frontal Gyrus (IFG), involved in action recognition and imitation; (b) the Inferior Parietal Lobule (IPL), which plays a role in integrating sensory information and understanding intentions; and (c) the Superior Temporal Sulcus (STS), important for perceiving biological motion and facial expressions. These areas work in concert to facilitate social interactions and cognitive empathy [83].

The positive response in multiple dimensions of autistic symptomatology supports the hypothesis of a global, rather than domain-specific, therapeutic effect. Furthermore, we cannot rule out time-dependent and time-of-day dependent effects of ELF-EMF treatments: application at different times (D protocol at night, AUT protocol in the daytime) could affect specific areas, thus improving treatment efficacy.

In more detail, in accordance with the work of Liboff [92,93,94,95] and Zhadin [96,97], ELF-EMF treatment at this range of intensity and frequency interacts with the earth’s magnetic field (GMF), generating ICR-like phenomena. ICR-like effects influence ion transport through modulation of membrane potential and regulation of Na^+^, K^+^, Ca^2+^, and Mg^2+^ fluxes. It cannot be ruled out that this leads to a neuronal excitability, resulting in optimization of synaptic transmission, with greater control of neuronal activation and a consequent effect of reducing the hyperexcitability characteristic of ASD.

Effects of ELF-EMFs are known to increase neurotransmitters synthesis (10 Hz on Dopamine and Serotonin20) and to interact with specific serotonergic receptors such as 5HT1 and 5HT2 [98]. As a confirmation for this potential effect, in a recent publication, agonist activity on 5HT1 was shown to produce improvements in treated ASD subjects [99]. Given the importance of serotonin circuits in both the development of ASD and related behavioral disorders [100] and the safety profiles of ELF-EMF treatment, further research could be crucial.

Finally, shifting the focus to the role of inflammation on ASD [8,9,10,11,12,13,14,15], it is important to note that several works have shown the activity of ELF-EMFs to modulate the inflammatory response (2 Hz effect of reducing IL-1β and TNF-α [59], 50 Hz effect of reducing IL-8 and increasing IL-10 [53,54,55], 75 Hz effect of increasing the activity of A2A and A3A receptors with anti-inflammatory effect [64]). It is therefore also likely that the effects on the CNS described above are associated with an indirect effect related to the modulatory activity of inflammation also at CNS level.

The limited sample (20 initial participants, with 4 dropouts) suggests the need for much larger studies to confirm these promising results, as well as to investigate in more detail the working hypotheses proposed in this paper.

## 5. Conclusions

Total body treatment with ELF-EMF of children with ASD has been shown to have a high safety profile and optimistic efficacy in reducing some of the symptoms associated with ASD. Performance improvements have also been observed, suggesting that ELF-EMF treatment could potentially become a very useful support in treating ASD symptoms. The effects appear to be direct on the CNS, as well as indirect via inflammatory response modulation. It will be important to expand the study sample in the near future, seeking to confirm current data and analyze these proposed mechanisms of action. Since ELF-EMF may modulate serotonin levels, increase choline uptake in the frontal cortex- hippocampus, and enhance vagal nerve activity, combining ELF-EMF with traditional pharmacological treatments may offer synergistic benefits. A coactive approach could further enhance therapeutic outcomes while minimizing side effects. The complementary mechanisms of action can target different aspects of ASD pathophysiology or improve neurotransmitter receptor sensitivity, complementing the effects of drugs.

## Figures and Tables

**Table 1:** Structure of ELF-EMF so-called D program. The program is configured as 9 combinations, called “Steps”. These are composed of emitted frequencies (measured in Hz), intensities (measured in μT), a delivery oscillation time (T-On), and no-field time (T-Off) (expressed in seconds), and an overall step duration time (measured in minutes).

Step	Frequency (Hz)	Intensity (μT)	T-On (s)	T-Off (s)	Duration (min)
**1**	1	20	5	2	20
**2**	2	20	5	2	20
**3**	3	20	5	2	20
**4**	1	20	5	2	20
**5**	2	20	5	2	20
**6**	3	20	5	2	20
**7**	1	20	5	2	20
**8**	2	20	5	2	20
**9**	3	20	5	2	20

**Table 2:** Structure of ELF-EMF so-called AUT program.

Step	Frequency (Hz)	Intensity (μT)	T-On (s)	T-Off (s)	Duration (min)
**1**	1	20	2	1	3
**2**	4	20	1	1	3
**3**	2	20	2	1	3
**4**	8	20	1	1	3
**5**	75	20	2	1	3
**6**	50	20	1	1	3
**7**	20	20	2	1	3
**8**	12	20	1	1	3
**9**	10	20	2	1	3

**Table 3:** Overview table of Achenbach Teacher Data for 1.5 to 5-year-olds in the study.

Test	T1 Mean	T2 Mean	*Stand. T. Stat.*	*p* Value
**Externalizing Area**	61.8	55.1	2.201	*0.028*
**Overall Area**	62.1	54.3	−2.383	*0.017*
**Withdraw Scale**	59.3	57.3	−1.784	*0.074*
**Attention Problems**	70.3	60.8	−2.366	*0.018*
**Aggressive Behavior**	57.9	54.0	−2.205	*0.027*
**Depressive Problems**	59.5	54.6	−1.983	*0.047*
**Oppositional Defiant Problems**	57.8	54.3	1.992	*0.046*
**Autism Spectrum Problems**	65.5	58.1	−1.897	*0.058*

**Table 4:** Overview table of Achenbach Teacher Data for 6 to 18-year-olds in the study.

Test	T1 Mean	T2 Mean	*Stand. T. Stat.*	*p* Value
**Internalizing Area**	58.6	44.6	2.366	0.018
**Externalizing Area**	55.3	48.1	2.214	0.027
**Overall Area**	57.0	46.9	−2.375	0.018
**Anxious/Depressed**	58.7	51.4	−2.201	0.028
**Withdrawn/Depressed**	58.1	51.4	−2.214	0.027
**Social Problems**	58.3	53.1	−2.023	0.043
**Attention Problems**	56.6	52.6	−2.201	0.028
**Thought Problems**	58.1	55.3	1.890	0.059

## Data Availability

http://www.sibeonline.com/pubblicazioni.html (accessed on 16 May 2024).
